# Prevalence, risk factors, clinical characteristics and treatment outcomes in patients with tinea auricularis: A retrospective study

**DOI:** 10.1016/j.jdin.2026.10.002

**Published:** 2026-10-12

**Authors:** Suthasanee Prasertsook, Charussri Leeyaphan, Sumanas Bunyaratavej, Pattriya Jirawattanadon, Lalita Matthapan, Waranyoo Prasong, Chatisa Panyawong, Akkarapong Plengpanich

**Affiliations:** Department of Dermatology, Faculty of Medicine Siriraj Hospital, Mahidol University, Bangkok, Thailand

**Keywords:** auricular dermatosis, dermatophyte, ear infection, fungal infection, tinea auricularis, *Trichophyton rubrum*, epidemiology, tinea facii

*To the Editor:* Tinea auricularis refers to a dermatophyte infection primarily localized to the auricular skin, distinguishing it clinically from adjacent tinea faciei or capitis. It frequently lacks typical annular plaques, often mimicking inflammatory conditions and leading to diagnostic delays.^1^ Because of its low reported incidence, comprehensive data on etiology and treatment remain limited.

This 6-year retrospective study at Siriraj Hospital, a tertiary care center in Thailand (2017-2022), evaluated 218 patients with suspected auricular involvement. Patients with confirmed tinea auricularis (*n* = 35, 16.1%; positive potassium hydroxide [KOH] examination with or without culture) and other dermatoses (*n* = 183) were analyzed (Table I). Immunocompromised status included diabetes, malignancy, HIV, or systemic immunosuppression. Continuous variables were analyzed according to data distribution. Exploratory multivariable regression assessed risk factors; outcomes (clinical and mycological cure) and recurrence were identified through chart reviews of routine follow-up and symptom-driven visits. For Fig 1, cumulative cure probability [1−S(t)] included 29 patients; 3 patients without cure were censored (6 excluded).

**Table I:** Factors associated with tinea auricularis infection in patients presenting with ear lesions

Factors	Ear lesion: *n* (%)		Crude odds ratio (95% CI)	*P* value	Adjusted odds ratio (95% CI)	*P* value
	Tinea *N* = 35	Other∗ *N* = 183				
Sex						
Female	18 (51.4)	116 (63.4)	1		-	
Male	17 (48.6)	67 (36.6)	1.64 (0.79-3.39)	.183	-	
Age: y ± SD	43.3 ± 18.2	51.5 ± 21.2	0.98 (0.96-1.00)	.035∗	0.98 (0.95-1.00)	.074
Having pets (*N* = 42)†	(*N* = 17)	(*N* = 25)				
No	8 (47.1)	14 (56.0)	1		-	
Yes	9 (52.9)	11 (44.0)	1.43 (0.42-4.93)	.569	-	
Immunocompromised						
No	20 (57.1)	148 (80.9)	1		1	
Yes	15 (42.9)	35 (19.1)	3.17 (1.48-6.81)	.002∗	3.48 (1.15-10.54)	.027∗
Symptom (*N* = 196)†	(*N* = 34)	(*N* = 162)				
Itch	34 (100.0)	155 (95.7)	-	.217	-	
Other	0	7 (4.3)			-	
Duration symptom: mo (IQR)	3 (1-12)	2 (1-12)	-	.683	-	
Ear involvement						
Bilateral	14 (40.0)	103 (56.3)	1		-	
Unilateral	21 (60.0)	80 (43.7)	1.93 (0.93-4.03)	.077	-	
Site of lesion (*N* = 170)†	(*N* = 25)	(*N* = 145)				
Postauricular	3 (12.0)	76 (52.4)	1		1	
Pinna	18 (72.0)	61 (42.1)	7.48 (2.10-26.56)	.002∗	8.80 (2.30-33.72)	.002∗
Preauricular	4 (16.0)	8 (5.5)	12.67 (2.40-66.94)	.003∗	11.77 (2.01-68.98)	.006∗
External canal involvement						
No	34 (97.1)	169 (92.3)	1		-	
Yes	1 (2.9)	14 (7.7)	0.36 (0.05-2.79)	.305	-	
Adjacent involvement						
None	15 (42.9)	115 (62.8)	1		1	
Face	17 (48.6)	38 (20.8)	3.43 (1.56-7.52)	.002∗	3.70 (1.21-11.13)	.022∗
Other	3 (8.6)	30 (16.4)	0.77 (0.21-2.82)	.689	1.03 (0.23-4.55)	.974

∗ Other auricular dermatoses (*n* = 183) comprised dermatitis not otherwise specified (*n* = 92, 50.3%), seborrheic dermatitis (*n* = 47, 25.7%), psoriasis (*n* = 11, 6.0%), contact dermatitis (*n* = 10, 5.5%), discoid lupus erythematosus (*n* = 5, 2.7%), atopic dermatitis (*n* = 3, 1.6%), and others (*n* = 15, 8.2%).

† Percentages for “Having pets”, “Symptom”, and “Site of lesion” were calculated based on the number of patients with available data because of missing chart documentation (n values for each subgroup are indicated in parentheses). Multivariable logistic regression used complete-case analysis (effective *n* = 170).

**Fig 1:**
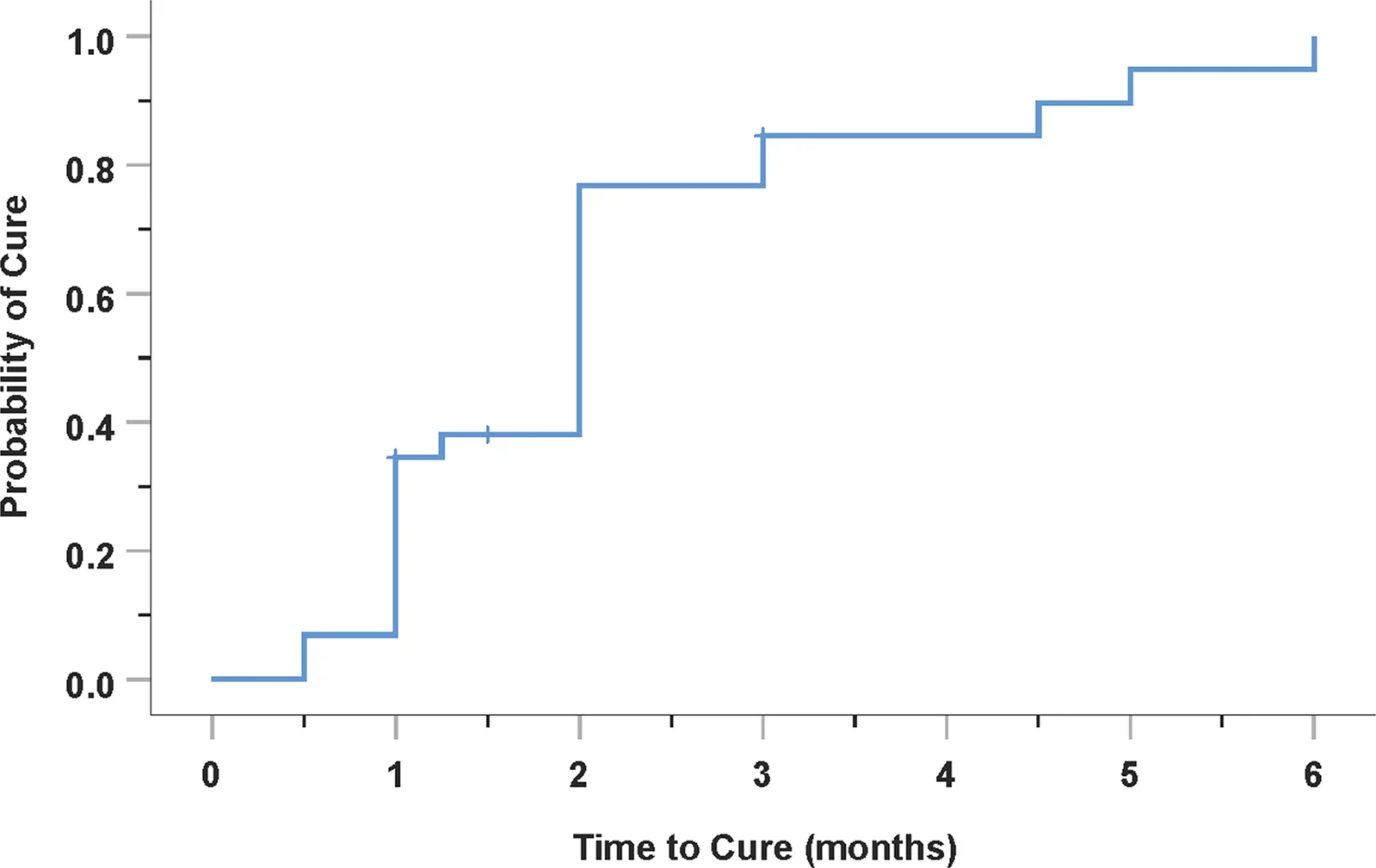
Kaplan-Meier curve showing the cumulative probability of mycological cure [1 − S(t)] over time among patients with tinea auricularis with documented follow-up (*N* = 29). Twenty-six patients achieved cure, and 3 patients without cure were censored at their last follow-up visit. (Six patients with no follow-up data were excluded from this analysis).

Mean age was 43.3 ± 18.2 years. Multivariable analysis identified immunocompromised status as a predictor of tinea auricularis (adjusted odds ratio [aOR], 3.48; *P* = .027). Lesion sites were mutually exclusive: pinna (aOR, 8.80; *P* = .002) and preauricular involvement (aOR, 11.77; *P* = .006) predicted tinea auricularis. Consistent with the literature, findings showed a significant association with facial involvement (aOR, 3.7; *P* = .022).^2^, ^3^ Among patients who underwent fungal culture (*n* = 18), KOH concordance was 88.9%. *Trichophyton rubrum* predominated (12/18, 66.7%), followed by the *Trichophyton mentagrophytes* complex (4/18, 22.2%). These findings provide regional data on geographic variation.

Concomitant onychomycosis occurred in 8.6% of patients, all of whom required systemic therapy, underscoring how concurrent infections influence treatment choice and duration. Furthermore, the association with immunocompromised status suggests that host immunity plays a more critical role in susceptibility than chronological age.^4^

Treatment included combination therapy (57.1%) and topical monotherapy (42.9%) using standard antifungals. The overall cure rate was 89.7% among the 29 evaluable cases (26/29). On a conservative whole-cohort basis, counting all 6 patients lost to follow-up as treatment failures, the cure rate was 74.3% (26/35). This high efficacy may be attributable to the absence of nondermatophyte molds. The median time to cure was 2 months (range, 0.5-6 months). Recurrence occurred in 26.9% (7/26) at 3 to 60 months after cure, predominantly among immunocompromised patients (57.1% of recurrent cases). Combination therapy was associated with higher observed cure rates in this retrospective descriptive analysis (100% vs 70%).

Limitations include the retrospective design and a tested sampling frame, introducing potential verification bias. The comparator group was highly heterogeneous, and the small number of tinea cases (*n* = 35) limits the robustness of our multivariable modeling, raising the possibility of chance findings. Microbiologically, incomplete culture data and the lack of molecular speciation or resistance testing preclude identification within the *T.* *mentagrophytes* complex (eg, *T. indotineae*).^5^ Lastly, the treatment evaluation is constrained by confounding by indication in nonrandomized treatment selection, unadjusted variation in drug class or duration, and potential attrition bias due to loss to follow-up.

In conclusion, tinea auricularis warrants high clinical suspicion and mycological confirmation. Prospective studies are needed to define optimal treatment.

### Declaration of generative AI and AI-assisted technologies in the writing process

None.

## Conflicts of interest

None disclosed.
